# Laughs and Laughter

**Published:** 1903-07

**Authors:** B. H. Colegrove


LAUGHS AND LAUGHTER.—Speaking of laughs, the most heart-rending laugh is the laugh of the summer girl that shook you, and is gadding around with some other fellow.

The most demoralizing laugh, to the married man, is the laugh of the family doctor when he comes up and slaps the married man on the back and shouts, "It's twins, old man!"

The most grotesque laugh is that of the fat woman, at a picnic, when she's got a pickle in her mouth.

The hollowest laugh is the laugh a man laughs when he sees the necktie his wife has bought him.

The cutest, sweetest little laugh is the laugh of your best girl. It generally costs a couple of theater tickets and a dollar or two hack-hire, though.

The most dangerous laugh is the laugh of a man when he's getting shaved at the barber shop.

The most untimely laugh is that of the boy who is making off in the darkness with the doped watermelon.

The most hilarious laugh is the laugh of the fellow who scoops in the stakes on a jack-high bluff.

One of the most comical laughs is that of a person who's got the hives, where the mouth sticks way out to one side and the nose acts as though it was mad about it.

The most enjoyable laugh is the laugh that's on someone else.

There are countless different kinds of laughter. Some people's laughter is soft and mellifluous, like the ripple of a meadow brook or the carol of the first robin of early spring, while other people's laughter reminds you of the screech of an old turkey gobbler, or the wild wail of an asthmatic donkey. A nice, clean-cut honest laugh is worth going a good many rods to hear. Next to having a good laugh ourself we like to hear a laugh like that.

Laughter is one of Nature's best medicines and beats pills and bitters out of sight. Laughter brushes down the cobwebs from the ceiling of the brain, dusts up and sweeps out the musty whims and cranky notions and gloomy forebodings, and adorns the chamber of thought with the beautiful picture of hope. When laughter enters the front door, despair scoots up the chimney-flue and remorse flies to the cat-hole. The phantoms of dread commence to dance funny break-downs, and the imps of gloom look like the members of a little German band.

Laughter works on the phrenic nerve which vibrates the diaphragm, thereby agitating the abdominal and thoracic regions, thus tending to establish an equitable blood supply and promoting the normal metabolism of the body.—*B. H. Colegrove in Diet. and Hyg. Gaz.*

